# Motor unit firing rates during spasms in thenar muscles of spinal cord injured subjects

**DOI:** 10.3389/fnhum.2014.00922

**Published:** 2014-11-14

**Authors:** Inge Zijdewind, Rob Bakels, Christine K. Thomas

**Affiliations:** ^1^Department Neuroscience, Medical Physiology, University Medical Center Groningen, University of GroningenGroningen, Netherlands; ^2^The Miami Project to Cure Paralysis, Departments of Neurological Surgery, Physiology and Biophysics, University of Miami Miller School of MedicineMiami, FL, USA

**Keywords:** motor unit recruitment, motor unit derecruitment, motor unit firing rate modulation, afferent input, motoneuron, persistent inward current

## Abstract

Involuntary contractions of paralyzed muscles (spasms) commonly disrupt daily activities and rehabilitation after human spinal cord injury (SCI). Our aim was to examine the recruitment, firing rate modulation, and derecruitment of motor units that underlie spasms of thenar muscles after cervical SCI. Intramuscular electromyographic activity (EMG), surface EMG, and force were recorded during thenar muscle spasms that occurred spontaneously or that were triggered by movement of a shoulder or leg. Most spasms were submaximal (mean: 39%, SD: 33 of the force evoked by median nerve stimulation at 50 Hz) with strong relationships between EMG and force (*R*^2^ > 0.69). Unit recruitment occurred over a wide force range (0.2–103% of 50 Hz force). Significant unit rate modulation occurred during spasms (frequency at 25% maximal force: 8.8 Hz, 3.3 SD; at maximal force: 16.1 Hz, 4.1 SD). Mean recruitment frequency (7.1 Hz, 3.2 SD) was significantly higher than derecruitment frequency (5.4 Hz, 2.4 SD). Coactive unit pairs that fired for more than 4 s showed high (*R*^2^ > 0.7, *n* = 4) or low (*R*^2^:0.3–0.7, *n* = 12) rate-rate correlations, and derecruitment reversals (21 pairs, 29%). Later recruited units had higher or lower maximal firing rates than lower threshold units. These discrepant data show that coactive motoneurons are drive both by common inputs and by synaptic inputs from different sources during muscle spasms. Further, thenar motoneurons can still fire at high rates in response to various peripheral inputs after SCI, supporting the idea that low maximal voluntary firing rates and forces in thenar muscles result from reduced descending drive.

## Introduction

Force is graded during voluntary contractions by the recruitment of motor units and by changes in motor unit firing rate (Monster and Chan, 1977). Since motor unit forces average 13% (6% SD) of maximal when their axons are stimulated at recruitment frequencies (5 Hz, Person and Kudina, 1972; Thomas et al., 1991), there is a large range over which force can be graded by changes in firing rate. After cervical spinal cord injury (SCI), recruitment becomes more important for force production in thenar muscles. Not only do thenar motor units generate nearly one third of their maximal force at recruitment frequencies (mean twitch/tetanic force ratio: 0.36, 0.11 SD; Håger-Ross et al., 2006), the firing rates achieved by motor units during maximal voluntary contractions are also low (mean 9.2 Hz, 3.1 SD; Zijdewind and Thomas, 2003). In uninjured subjects, maximal thenar motor unit firing rates are about three times higher, averaging 34.1 Hz (10.2 SD; Thomas, 1997). Two findings suggest that these low maximal motor unit firing rates after SCI result from reduced descending drive rather than the inability of motoneurons to respond to various inputs. First, the firing rate of one thenar motor unit was higher during a spasm (involuntary muscle contraction) than during a maximal voluntary contraction (Zijdewind and Thomas, 2003). Second, maximal motor unit firing rates could also be increased by combining a maximal voluntary contraction with an evoked muscle spasm (Zijdewind et al., 2012).

Here, our aim was to record the firing behavior of different thenar motor units during muscle spasms. If the activity of two units was correlated during a muscle spasm, the motoneurons had likely responded to an external, synaptic drive. However, if the firing behavior of unit pairs was not correlated, synaptic inputs may have activated different parts of the motoneuron pool or intrinsic currents may have affected the firing of one motoneuron more than the other. Strong increases in motor unit firing rates during spasms would also support the idea that deficits in descending drive to the thenar motor pool limit maximal voluntary motor unit firing rates and force after cervical SCI.

## Methods

### Subjects

Data were recorded from seven individuals with chronic (>1 year) cervical SCI (1 woman, 6 men; aged 20–53 years) due to a fall (*n* = 1) or a motor vehicle (*n* = 2), bicycle (*n* = 1) or diving accident (*n* = 3) that occurred from 1–26 years ago. Injury level was at C4 (*n* = 1), C5 (*n* = 2), C6 (*n* = 2) or C7 (*n* = 2) and was classified as A (*n* = 5) or B (*n* = 2) using the American Spinal Injury Association impairment scale. The thenar muscles of the hand more likely to contract involuntarily were studied once in each subject (*n* = 5 left; *n* = 2, right). Two subjects had some voluntary control of their thenar muscles. The University of Miami Institutional Review Board approved all of the experiments and each subject gave written informed consent to participate.

### Setup for thenar motor unit and muscle measurements

Each subject sat in their wheelchair with the test arm supported in a vacuum cast. The hand lay in modeling clay with the palm up, and was held in place by a metal plate and Velcro straps (Thomas, 1997). Electromyographic activity (EMG) was recorded from the distal and proximal muscle surfaces using wire electrodes taped across the muscle. The distal electrode lay across the interphalangeal joint, the proximal electrode was placed at the base of the thenar eminence, and a common electrode lay across the middle of the muscle bellies (Westling et al., 1990). Potentials from single motor units were recorded intramuscularly using a custom-made tungsten microelectrode. A transducer was aligned with the thumb to measure abduction and flexion forces at right angles. Resultant force was calculated from these forces.

### Protocol

Spasms were triggered by shoulder movements, by lifting and dropping one leg on the foot plate of the wheelchair, or they occurred spontaneously. Between spasms (≥1 min), the intramuscular electrode was moved in order to sample different motor units. Maximal thenar muscle force was evoked by stimulating the median nerve just proximal to the wrist for 1 s at 50 Hz using supramaximal pulses (20–50% higher than the intensity that evoked a maximal compound muscle action potential). The force generated during spasms was normalized to the respective 50 Hz force for a given muscle to enable comparison of data across spasms and subjects.

### Data collection and analysis

Intramuscular EMG, surface EMG, and force were sampled online at 12,800 Hz, 3,200 Hz, and 400 Hz, respectively, using a SC/Zoom system (Umeå University, Sweden). Potentials belonging to the same motor unit were identified by amplitude, duration and shape, and verified by overlaying all of the marked potentials for a given spasm. The time between the potentials of a single motor unit was converted to instantaneous frequency by using the reciprocal of the interspike intervals (ISI). For each spasm, firing frequency was determined: (1) when the motor unit was recruited (first ISI shorter than 500 ms; Fuglevand et al., 2006); (2) at 25%, 50%, 75% and 100% maximal force during the increase in spasm force (an average over three consecutive ISIs was calculated for each measure); and (3) unit derecruitment (last ISI less than 1000 ms) during the decrease in spasm force.

When pairs of units were coactive during spasms, estimates were made of the amplitude of persistent inward currents boosting the effective synaptic drive by calculating the change in firing frequency of the first recruited unit (control unit, CU) when the second unit (test unit, TU) was recruited vs. derecruited (ΔF = frequency CU at recruitment of TU- frequency CU at derecruitment of TU; Kiehn and Eken, 1997; Bennett et al., 2001; Gorassini et al., 2004). In cat motoneurons, it has been suggested that the persistent calcium current activates with a time constant of about 50–100 ms (Lee and Heckman, 1998; Venugopal et al., 2011). In order to allow full activation of persistent inward currents in the CU, we selected control and TUs that were recruited at least 1000 ms apart. To measure whether a unit pair was influenced by common drive, the mean firing frequency of each unit was calculated over consecutive 500 ms epochs. Data from simultaneously active units were plotted against each other and the correlation coefficient of the linear-regression analysis was calculated. For the paired unit analysis only unit pairs with an overlap of eight 500 ms-epochs (4 s) and a correlation coefficient (*R*^2^) above 0.7 were used.

Surface EMG was rectified, integrated every 250 ms, and plotted against the resultant force for the rising phase of the spasm and separately for the decline. The association between EMG and force values was determined using linear regression analysis.

### Statistics

Mean (SD) data are given. Relationships between surface EMG and force, recruitment and derecruitment frequency, maximal frequency and maximal force, and maximal frequency of the first and second recruited units used for paired analysis were all analyzed using least squares linear regression. Differences in rate of force development and decline during the upward and downward phase of a spasm were tested with a multilevel analysis (mixed model analysis, IBM SPSS 22) with force rate as the first level and the muscle status (residual voluntary control or not) as the second level variable. In the analysis the intercept of the relation between the rate of force change and muscle status was first modeled as a fixed variable. Including a random intercept improved our model significantly, but including a random slope made no further improvement (−2 log likelihood did not change significantly).

Motor unit firing rates were compared at recruitment, derecruitment and at different force levels. Differences in motor unit firing rates were analyzed using a multi-level analysis so all data were included in the analysis (not all units could be followed or were not active across all force levels). The data were modeled in a hierarchical multi-level model with unit firing rate as the lowest level variable. To identify that the data came from a specific motor unit and subject, higher level group-identifiers were included. We first assessed possible differences in firing rates between subjects with or without residual voluntary force (muscle status). No significant differences were observed in recruitment, maximal or derecruitment frequencies. Therefore frequency data were pooled for all subjects. Differences in firing rate were examined at specified force levels. In the analysis the intercept of the relation between firing frequency and force level was first modeled as a fixed variable. Including a random intercept improved our model significantly. Including a random slope, however, did not improve our model any further (−2 log likelihood did not change significantly). Therefore, only the statistical analysis including the random intercept was presented in the Results.

## Results

**Figure 1:**
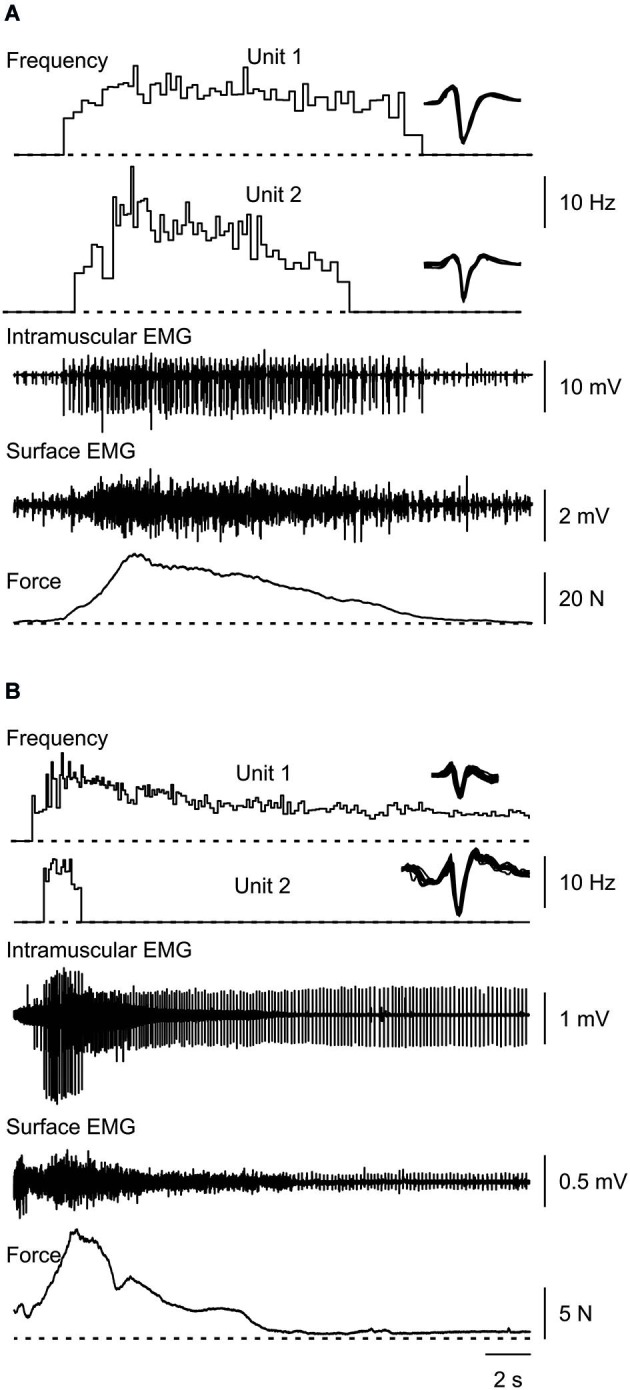
**Typical muscle spasms showing two motor units that are recruited, increase their firing rate during the force rise, decrease firing rate as the force declines, and are derecruited (A,B) or continue to fire after the contraction (B)**. Overlays of the marked potentials show accurate identification of each motor unit (right).

**Figure 2:**
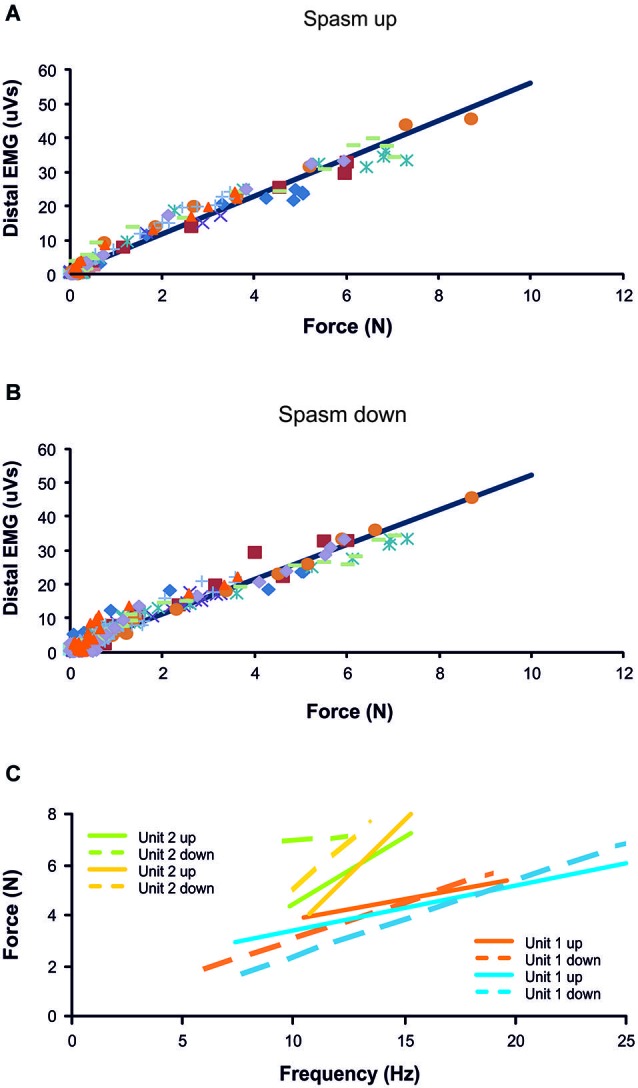
**Distal EMG-force relationship during the force rise (A; *R*^2^ = 0.97) and fall (B; *R*^2^ = 0.95) of 10 spasms (different symbols) recorded from one subject. (C)** Frequency-force relationship for 2 units, each followed during the force increase and decrease of two different spasms (*n* = 4 spasms total). Data are from the same subject as in **(A)** and **(B)**.

Most thenar muscle spasms occurred spontaneously or spasms were evoked by movement of a shoulder or leg. The mean increase in spasm force lasted 4.17 s (SD, 1.91) while the force decrease lasted 6.50 s (SD, 5.69 s, *n* = 37 spasms). Two other spasms lasted up to 24 s because they had double peaks. Possibly two spasms had occurred in close succession. The rate of change for EMG and force was similar during the upward phase of a spasm (1.89 N/s, SD 3.02) and during the downward phase (1.59 N/s, SD 2.95, *F* = 6.635, *P* = 0.58, Figure 1). In general, mean EMG and force were strongly associated during the spasms (Figures 2A,B; up: 32/39 spasms, *R*^2^ > 0.69; down: 32/39 spasms, *R*^2^ > 0.52). Some motor units were not derecruited following spasms but rather continued to fire for several minutes at low firing rates (Figure 1B).

### Motor unit firing rate increases during spasms

**Figure 3:**
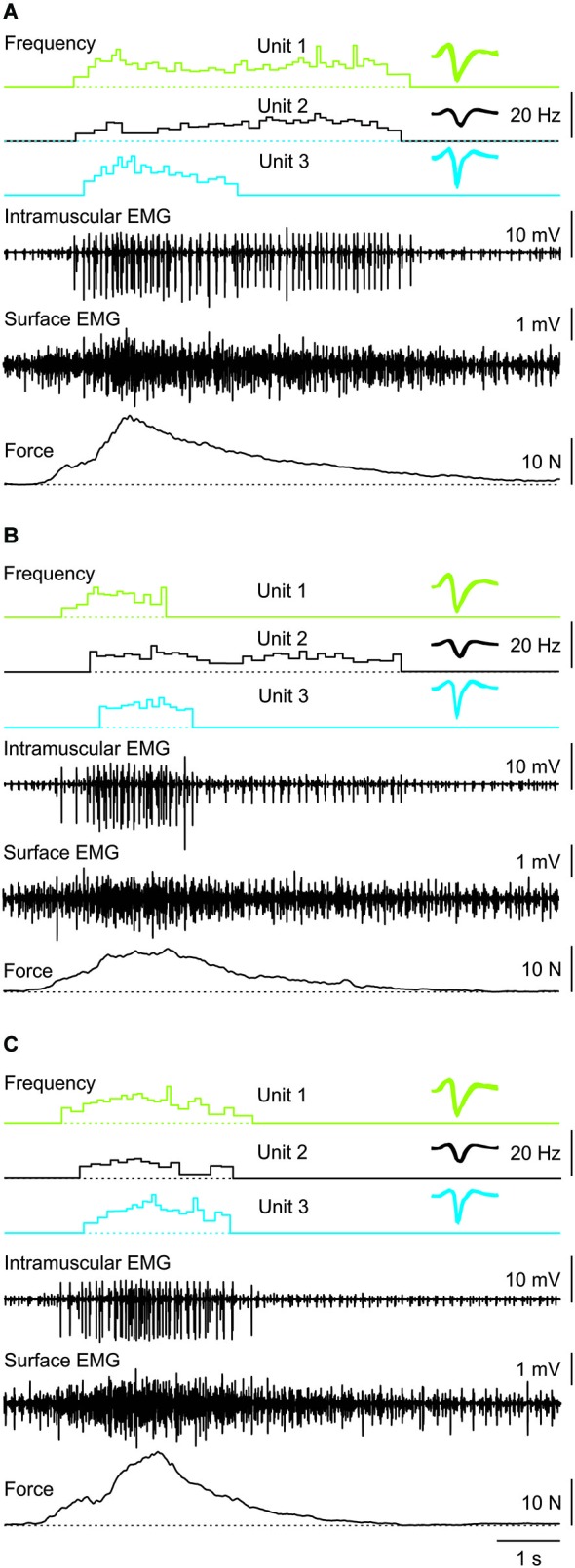
**Three consecutive muscle spasms from one subject in which three coactive units behaved differently despite similar rates of force increase and decrease during each spasm**. Recruitment order was always Unit 1, 2, then 3. Unit derecruitment occurred in the reverse order in two spasms **(A,C)** but was disordered (Unit 1, 3, 2) in the other spasm **(B)**. Overlays of the marked potentials show accurate identification of each motor unit (right).

The maximal force obtained during spasms averaged 7.5 N (7.3 SD) or 39.5 % (33.2 SD) of the 50 Hz force. We could track 72 motor units throughout or during part of a spasm (Figures 1, 3). The recruitment force of these units varied between 0.04–23.8 N (mean 2.2 N, 3.0 SD) or 0.2–103.1% of 50 Hz force. Thus, both low and high threshold units were analyzed.

Motor unit firing rate at recruitment averaged 7.1 Hz (3.2 SD, *n* = 66 units) and increased to a mean maximal firing frequency of 11.6 Hz (4.4 SD, *n* = 64 units, *P* < 0.001; Figure 5) even though the force was often submaximal (<50 Hz force). At derecruitment, motor unit firing rate (5.4 Hz, 2.4 SD, *n* = 66) was significantly lower than both the recruitment and maximal firing rates (both *P* < 0.001). Of these units, 41% (25/61) had higher firing rates at derecruitment than at recruitment in at least one spasm (see e.g., Unit 1 in Figure 3B).

**Figure 4:**
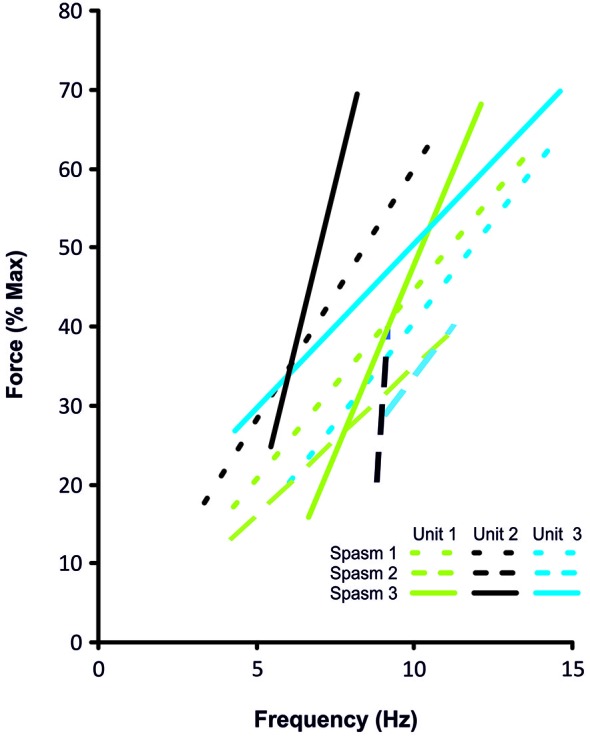
**Frequency-force relationship for the 3 coactive units during the force increase of spasm 1, 2 and 3 (dotted, dashed, solid lines, respectively) shown in Figure 3**.

**Figure 5:**
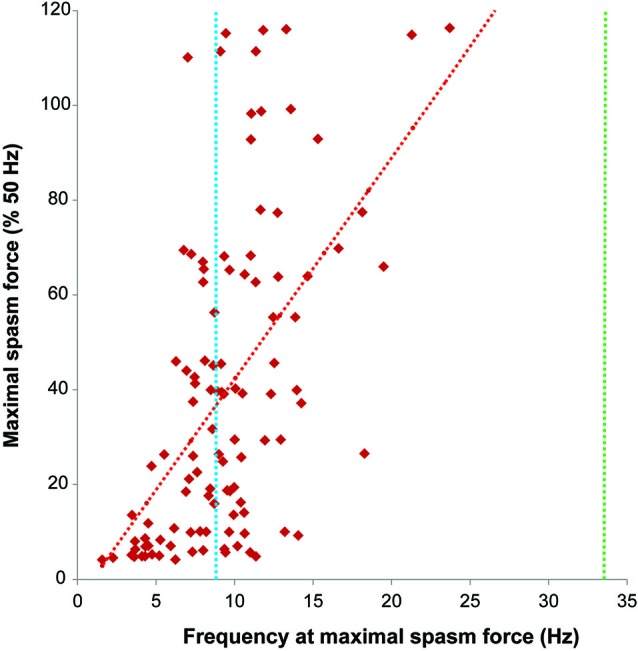
**Firing rate (Hz) at maximal spasm force vs. maximal spasm force (% 50 Hz force)**. The red interrupted line shows the least squares regression line (*R*^2^ = 0.30). The dotted lines indicate the average firing rates of motor units during maximal voluntary contractions of the thenar muscles in subjects after spinal cord injury (SCI) (9.2 Hz, blue; Zijdewind and Thomas, 2003) and in able-bodied subjects (34.1 Hz, green; Thomas, 1997).

**Figure 6:**
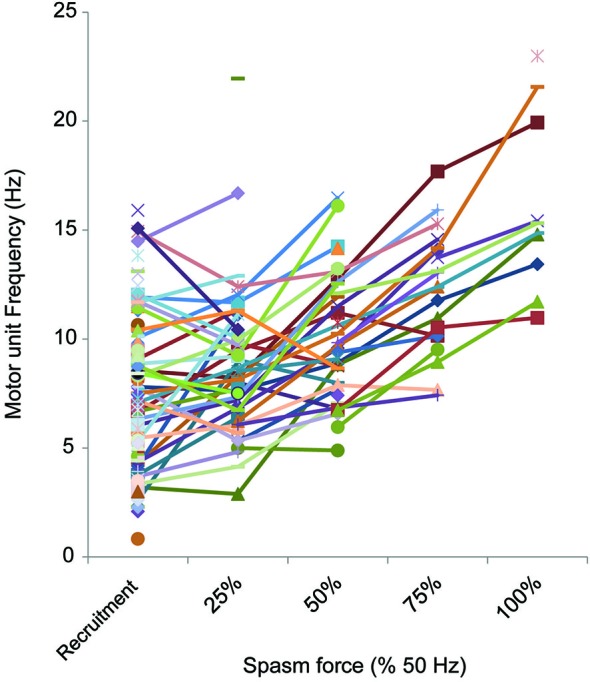
**Motor unit firing rate at different force levels (% 50 Hz force)**. The firing rate of motor units (reciprocal of the mean of 3 interspike intervals) at each 25% force step was plotted, starting at recruitment. Not all spasms attained the force evoked by 50 Hz stimulation or units could not be followed up to maximal force.

Motor units that could be followed during stronger spasms (≥25% maximal force) showed clear rate modulation (Figure 6). The average firing rate of units that were active at 25% of maximal (50 Hz) force was 8.8 Hz (3.3 SD, *n* = 27), at 50% was 10.0 Hz (2.9 SD, *n* = 24), at 75% was 12.2 Hz (2.8 SD, *n* = 16) and at 100% was 16.1 Hz (4.1 SD, *n* = 10; Figure 6).

Motor units did not necessarily behave similarly in repeat spasms. Figure 2C shows one unit behaving similarly during repeat spasms (Unit 1) while the firing behavior of the other unit differed (Unit 2). Other units were coactive during the same spasm and could be followed during multiple spasms (Figure 3). All three units co-modulated their firing rate during the force increase of the first spasm (dotted lines), whereas each unit behaved differently during the second (dashed lines) and third spasms (solid lines; Figure 4).

### Maximal firing rates of later recruited units were either higher or lower than earlier recruited units

**Figure 7:**
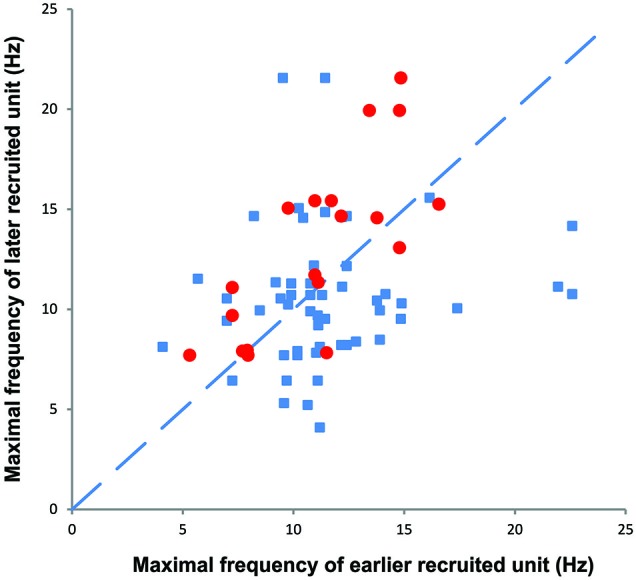
**The maximal firing frequency of all motor unit pairs obtained during spasms (blue)**. Later recruited units had lower, similar, or higher maximal firing rates than earlier recruited units. The red symbols refer to motor unit pairs that display a moderate (*R*^2^ > 0.3) to high rate-rate correlation during spasms.

During voluntary contractions, coactive units can display similar rate modulation (common drive; e.g., De Luca and Hostage, 2010) or low threshold units can attain lower maximal firing rates than later recruited units (e.g., Moritz et al., 2005; Oya et al., 2009). When more than one unit could be tracked throughout a spasm (e.g., Figures 3, 7), there was no systematic difference between the maximal firing rates of the later and earlier recruited units. That is, the maximal firing rate of the later recruited units could be either higher or lower than that recorded for earlier recruited units (Figure 7).

### Paired unit analysis

During spasms, 23/92 unit pairs (25%) had a moderate rate-rate correlation (*R*^2^ > 0.3), but only 8 pairs showed a high rate-rate correlation (*R*^2^ > 0.7). Only 24 of the 92 pairs fired simultaneously for more than 4 s. Of the 24 pairs, 12 pairs (50%) showed a moderate rate-rate correlation, but only 4 pairs showed a high correlation (*R*^2^ > 0.7; during voluntary contractions in control subjects mean *R*^2^ = 0.78, Mottram et al., 2009). Each of these 4 units pairs has similar recruitment forces so none of these pairs fulfilled our criterion a recruitment being separated by at least 1000 ms. Thus, none of our unit pairs (coactivated *involuntarily* during spasms) were suitable for the conventional paired unit analysis to estimate the amplitude of the persistent inward current (Gorassini et al., 2004).

Of the 72 unit pairs for which we could determine derecruitment, 21 pairs (29%) showed reversals of derecruitment. That is, the unit that was recruited first was also derecruited first (Figure 3B). Eleven unit pairs were followed through multiple spasms (range: 2–4). During a total of 26 repeat contractions, there were two recruitment reversals (7.7%) and 10 derecruitment reversals (38.5%).

## Discussion

The main finding of the present study was distinct rate modulation of motor units during thenar muscle spasms in individuals with SCI. These data confirm in a larger group of motor units that thenar motoneurons impacted by SCI are still capable of firing at higher frequencies in response to various peripheral inputs, as are motoneurons innervating limb muscles (Thomas and Ross, 1997; Gorassini et al., 2004). Although most thenar spasms were submaximal in terms of unit firing rate and force, this rate modulation is an important contributor to spasm strength. Paralyzed thenar motor units generate 46% and 72% of their maximal force at 7.1 Hz and 16.1 Hz, the average recruitment and maximal firing rates during spasms, respectively (Håger-Ross et al., 2006). Further, the low or high correlations in rate-rate plots, and the lower or higher maximal firing rates of earlier vs. later recruited units suggest that motor units activated during muscle spasms are not always driven solely by common inputs.

### Higher motor unit firing rates during thenar spasms than voluntary contractions

The mechanisms underlying muscle spasms are still not completely understood. After SCI it is thought that constitutively active neuromodulatory receptors (Murray et al., 2010) and long duration excitatory post synaptic potentials (Norton et al., 2008) act to increase the amplitude and the likelihood of activation of persistent inward currents in motoneurons (Button et al., 2008). These changes and reduced inhibition after SCI make motoneurons easier to activate from peripheral inputs (Gorassini et al., 2004; Norton et al., 2008), and thus evoke long-lasting firing (Zijdewind and Thomas, 2003, 2012). Overall, the peripheral inputs to motoneurons (amount, strength, source) and their effects on motoneurons seem to increase after SCI whereas the amount, strength, and effects of descending input can be dramatically reduced (Lemon, 2008; Oudega and Perez, 2012; Thomas et al., 2014). The mean maximal motor unit firing rate was low during spasms (16.1 Hz) compared to the firing rate recorded during maximal voluntary contractions performed by able-bodied subjects (34.1 Hz; Thomas, 1997) or the stimulation frequency needed to evoke maximal force in thenar motor units paralyzed by SCI (30–50 Hz; Håger-Ross et al., 2006). Nevertheless, the thenar motor unit firing rate increased significantly more during spasms than previously seen during maximal voluntary contractions of thenar muscles performed by SCI subjects (Zijdewind and Thomas, 2003). In the latter paper, the units were recruited at 5.7 Hz (2.5 SD) and reached 9.2 Hz (3.1 SD) at maximal voluntary force. When units were recruited at low force levels and monitored up to maximal force they showed little or no rate gradation. The maximal increase in unit firing rate from 25% to 100% maximal voluntary force was only 4.2 Hz and some units actually showed a decrease in rate (maximal decrease 2.5 Hz; Zijdewind and Thomas, 2003; Figure 2). Taken together, these results indicate that motor units are not driven maximally during thenar voluntary contractions following SCI.

The combination of voluntary input and weak spasms indeed resulted in higher unit firing rates than did voluntary input alone (13.4 vs. 11.7 Hz; Zijdewind et al., 2012), stressing the importance of peripheral input for increasing firing rates of thenar motoneurons after SCI. In contrast, triceps brachii motor units often fired at high rates during maximal voluntary contractions after SCI (range: 6–54 Hz, for review see, Thomas et al., 2002; Johanson et al., 2013), differences that may relate to stronger monosynaptic corticospinal input to motoneurons that supply hand vs. limb muscles.

### Paired motor unit behavior

The short duration and relatively fast rate of force change during naturally occurring spasms makes it difficult to use the paired unit analysis to estimate persistent inward current amplitudes (Kiehn and Eken, 1997; Bennett et al., 2001; Gorassini et al., 2004; however, cf. Revill and Fuglevand, 2011). First, most of our unit pairs were activated within a short time window (<500 ms), which suggests that these units were activated by the same trigger. Second, most of our unit pairs were coactive for short periods. Third, only a few unit pairs showed a high rate-rate correlation which suggests that after recruitment these units received different inputs or modulated their firing on the basis of intrinsic motoneuron properties. The present data illustrate how difficult it is to use the paired unit method when *both units are activated during a spasm*. In a previous paper (Gorassini et al., 2004) these paired unit methods were useful because contraction duration and recruitment separations were controlled. The CU was steadily activated voluntarily before the spasm and compared to a TU that was only activated during the spasm.

Nevertheless, these persistent currents probably underlie the prolonged motor unit firing that occurs after some spasms (Figure 1B) and the lower mean frequency of individual motor units at derecruitment (5.4 Hz, SD 2.4) vs. recruitment (7.1 Hz, SD 3.2). In contrast, recruitment and derecruitment frequencies did not differ significantly when SCI subjects activated thenar units voluntarily (recruitment: 5.6 Hz, SD 2.7; derecruitment: 5.2, SD 2.0; Zijdewind and Thomas, 2003). Whether this observation reflects larger persistent inward currents during spasms than during voluntary activation is unclear.

Motor unit derecruitment reversals (first recruited unit also stops firing first) were observed in 29% of the motor unit pairs during spasms, which is comparable to the percentage found in units left under voluntary control after injury (25%; Zijdewind and Thomas, 2012) but lower than the reversal percentage for unit pairs that were spontaneously active (56%; Zijdewind and Thomas, 2012). The derecruitment reversals, and the higher motor unit firing frequencies at derecruitment vs. recruitment probably indicate differences in input across motoneurons. It is known that inhibition effectively closes ion channels involved in persistent currents (Hyngstrom et al., 2008). Thus, an uneven distribution of inhibition over the motoneuron pool could result in inhibition and/or derecruitment of some motoneurons but not others.

### Maximal firing rates of later recruited units are higher or lower than those of earlier recruited units

In able-bodied subjects, motor units often show an association between recruitment threshold and mean motor unit firing rates but these relationships can differ. In some studies, motor units recruited at higher forces fire at a lower rate than units activated earlier at weaker forces; i.e., an onion skin profile (e.g., De Luca and Hostage, 2010). This theory of common drive relies upon variations in motoneuron properties (e.g., input resistance) to account for recruitment of motoneurons in the same pool and assumes that these neurons receive shared synaptic drive resulting in comparable firing rate modulation. However, this explanation does not take into account other variations in motoneuron properties (e.g., differences in input-output relations; i.e., gain, amplitude and kinetics of persistent currents, Lee and Heckman, 1998), the importance of which is suggested by data that show earlier recruited units reach lower maximal firing rates than later recruited units (e.g., Moritz et al., 2005; Oya et al., 2009).

During spasms there was no systematic relationship between the maximal firing rate of earlier and later recruited units (Figure 7), even for unit pairs in which the rate-rate correlation was significant. Further, some units that showed significant co-variation in firing rate still had lower correlation coefficients than reported for units from control subjects during voluntary contractions (*R*^2^ = 0.78; Mottram et al., 2009). This behavior suggests that during a spasm some motoneurons share common input that results in similar firing rate modulation. However, at the same time, simultaneously active motoneurons in the same pool probably also have different input-output relations (Kernell and Hultborn, 1990) or receive different amounts and/or sources of synaptic input to give uncorrelated data. Not only may this result in different unit firing rates and durations, but also in derecruitment reversals. In Figure 3, Unit 1 was recruited first in all three spasms but was derecruited last in two spasms and first in the other despite similar rates of force development and decline across spasms.

### Functional implications

The present data show the importance of various afferent inputs for motor unit rate modulation after SCI. However, excitatory input from the periphery seems less focused and distributed to a wider range of motoneuron pools after SCI (Hyngstrom et al., 2008; Johnson et al., 2013) thereby increasing the chance of activating multiple muscles. The uncontrolled movements that can result are one reason why spasms are considered a negative consequence of SCI. Thus, medication is often prescribed to reduce the number and strength of the spasms by reducing the afferent input and/or motoneuron excitability *per se*. Chronic baclofen, however, weakens motor units (Thomas et al., 2010) so non-pharmacological ways to manage spasticity may allow the high motor unit firing rates typical of some spasms to maintain muscle strength and to reduce atrophy to some extent (Kernell et al., 1987; Ditor et al., 2004; Harris et al., 2007). Medication will also impact the function of muscles that remain under voluntary control after SCI so these situations reflect the difficult choices that have to be made to manage muscle spasms.

## Author contributions

Zijdewind: Project conception and design, data acquisition, analysis and interpretation, manuscript writing, final approval of the manuscript.

Rob Bakels: Data analysis and interpretation, manuscript writing, final approval of the manuscript.

Christine K. Thomas: Project conception and design, data acquisition, analysis and interpretation, manuscript writing, final approval of the manuscript.

## Conflict of interest statement

The authors declare that the research was conducted in the absence of any commercial or financial relationships that could be construed as a potential conflict of interest.
